# Fear of recurrence in elderly patients with coronary heart disease: the current situation and influencing factors according to a questionnaire analysis

**DOI:** 10.1186/s12872-022-02853-w

**Published:** 2022-09-21

**Authors:** Jing Zhen, Jing Wang, Yi-Lin Wang, Jin Jiao, Jing Li, Xiao-Jing Du, Yan-Ling Li

**Affiliations:** 1 Office of Infection and Control, Affiliated Hospital of Hebei University, Baoding, China; 2 Inspection department, Affiliated Hospital of Hebei University, Baoding, 071000 Hebei China; 3 School of Basic Medicine of Shandong University, Jinan, 250012 Shandong China; 4 Medical Oncology Department, Affiliated Hospital of Hebei University, Baoding, China; 5 Cardiovascular Department, Affiliated Hospital of Hebei University, Baoding, China; 6 School of Nursing, He Bei University, Baoding, China; 7 Department of Tuberculosis, Affiliated Hospital of Hebei University, Baoding, China

**Keywords:** Coronary heart disease, Elderly patients, Fear of recurrence, Interventions, Social support

## Abstract

**Objective:**

Fear of recurrence is a common psychosocial sequela among patients with heart disease. Analyses of coronary heart disease, particularly in elderly patients, are relatively rare. This study aimed to investigate the current situation in this context, as well as the influencing fear factors concerning recurrence in elderly patients with coronary heart disease.

**Methods:**

A total of 200 elderly outpatients with coronary heart disease were recruited to participate in this survey from a tertiary hospital in Baoding (China). The questionnaires included items from the Disease Progression Simplified Scale, the Simplified Coping Style Questionnaire, and the Social Support Rating Scale (SSRS). Univariate and multivariate regression analyses were adopted to investigate the influencing factors on the fear of recurrence.

**Results:**

The fear of recurrence score in elderly patients with coronary heart disease was (38.46 ± 8.13), among which 119 cases (59.5%) scored higher than 34 points. The SSRS total average score was (34.89 ± 9.83) points. Positive coping style and social support were negatively correlated with the total score of recurrence fear (r =  − 0.621, − 0.413, both *P* < 0.001). There was a positive correlation between negative coping style and the total score of recurrence fear (r = 0.232, *P* < 0.001). Multiple linear regression analysis showed that the course of the disease, the number of disease recurrence cases, active coping, and social support were relevant factors in fear of recurrence (all *P* < 0.05).

**Conclusion:**

The detection rate of fear of recurrence in elderly patients with coronary heart disease was relatively high but could be reduced by active interventions and enhancing social support.

## Introduction

The ‘Healthy China 2030’ planning outline incorporated the prevention and treatment of major chronic diseases into the Healthy China initiative [1]. Among all the major chronic diseases, coronary heart disease (CHD) is the most common underlying illness and a leading cause of death among the elderly with chronic persistent. European and American prevention guidelines showed that patients with clinically evident cardiovascular disease were considered to be at high risk of recurrence and should receive a stronger focus in clinical treatment. [2] Despite comprehensive efforts to optimise patient care and manage individual risk, recurrent cardiovascular events remain a major concern among patients with CHD, particularly in elderly patients with more complex and severe clinical symptoms compared to younger patients [3].

Recent studies have shown that the fear of recurrence or disease progression is one of the most common unmet needs and concerns among cancer survivors, and clinically significant effects often accompany these fears, thereby influencing their quality of life. [4, 5] ‘Fear of recurrence’ is defined as the fears and concerns related to disease recurrence or progression that can arise at the time of diagnosis and persist throughout the patient’s survival trajectory. [6] The cumulative risk of recurrent CVD events is as high as 10–12% in the first year and 30–40% over five years [7]. In multivariate analyses, advanced age persisted as a significant independent risk factor for reinfarction or fatal CHD. The risk ratio for recurrence was 0.91 in the 30–69-years-of-age group but rose to 1.38 in the 70–79-years-of-age group*.* [8] This finding indicates that most elderly patients will eventually experience several relapses and/or progression, which can cause concern and anxiety about the recurrence of CHD events.

The fear of CHD recurrence is considered a major concern among CHD survivors and is associated with post-traumatic stress disorder, inferior prognosis, lower levels of well-being, and a worse quality of life worldwide [9]. These aspects are mainly manifested in the patient's over-examination, over-alertness, and over-concern about changes in their physical symptoms, and taking some physical symptoms (e.g. pain and chest tightness) as signs of aggravation, which will increase the patient's psychological burden in severe cases and can even lead to anxiety and depression [2], thereby negatively affecting physical function and social life and resulting in a low quality of life [10].

Most domestic studies on the fear of disease recurrence currently focus on cancer patients [11–13]. Few studies involving CHD patients in this regard have been conducted to date, particularly involving elderly CHD patients with a high disease recurrence rate. Accordingly, this study aimed to explore the current situation related to fear of disease recurrence in elderly CHD patients and analysed its influencing factors to provide a basis for further intervention and prevention of disease recurrence.

## Participants and study method

### Research participants

A total of 200 elderly CHD patients who were followed up in the outpatient department of cardiology of a tertiary hospital in Baoding were recruited from March 2020 to February 2022. All patients were clinically diagnosed by invasive coronary angiography (ICA), a gold standard test for ensuring high accuracy [14]. The authors also collected the patients’ electrocardiogram and cardiac ultrasound examination results for auxiliary diagnosis.

The study’s inclusion criteria were as follows: (1) patients aged ≥ 60 years old; (2) clinically diagnosed with coronary atherosclerotic heart disease, i.e. > 70% stenosis of at least one main coronary artery induced by atherosclerosis; (3) patients had no cognitive impairment and normal communication skills; (4) all patients provided informed consent for inclusion in the study.

The study’s exclusion criteria were as follows: (1) patients with valvular or congenital heart diseases; (2) patients with severe infection anywhere in the body; (3) patients with severe liver and kidney system diseases and a history of tumours; (4) patients with systemic immune system and connective tissue diseases [15]. This study was demonstrated by the ethics committee of the relevant institution.

### Research method

#### Research tools

##### General information questionnaire

The questionnaire was designed by the researchers and included items concerning age, gender, marital status, educational level, personal monthly income, previous occupational role/s, disease course, number of disease recurrences, medical insurance, and knowledge of the disease.

##### Fear of progression questionnaire–short form

The Fear of Progression Questionnaire–Short Form (FoP-Q-SF) was developed by Mehnert et al. [16] in 2006 and is mainly used to assess the level of fear of disease recurrence in patients with cancer and chronic diseases. The FoP-Q-SF was translated into Chinese and revised by Wu Qiyun et al. [17], including two dimensions of physical health and social family (total, 12 items). Each item was scored on a 5-point Likert scale from ‘never’ to ‘always’, respectively, from 1–5 (total score, 12–60). A higher score indicated a stronger fear of disease recurrence; a total score higher than 34 indicated a defined level of clinical significance. The Cronbach's alpha (α) coefficients for the FoP-Q-SF and two dimensions were 0.885, 0.834, and 0.806, respectively, with good reliability and validity.

##### Simplified coping style questionnaire

The self-reporting scale was a Chinese version of the Simplified Coping Style Questionnaire (SCSQ), revised by Jie Yaning [18]. The scale included 20 items in total to measure individual coping styles, including two subscales, i.e. positive (12 items) and negative (8 items) coping. Positive coping reflected the level of positive coping styles, such as ‘solving problems through work, study or other activities’, or ‘seeing the good in things’. Contrastingly, negative coping reflected the level of negative coping styles, e.g. drinking/smoking to forget about problems or relying on others to solve these problems [19]. The scale adopted a 4-point Likert scoring system. Each item was scored from 0 to 4 points (0 = never, 1 = seldom, 2 = often, 3 = always), and the results were the average scores of the positive and the average score of the negative coping dimensions, respectively. Higher scores on each subscale reflected the level of the coping style. The Cronbach's α coefficient of the scale was 0.901, and the test–retest reliability was 0.892, indicating good reliability and validity.

##### Social support rating scale

The Social Support Rating Scale (SSRS) [20] included three dimensions, i.e. objective support (3 items), subjective support (4 items) and support benefit expenditure (3 items) (total, 10 items), and the total score ranged from 12 to 66 points. A higher score indicated a higher level of social support. The scale’s Cronbach's α coefficient was 0.896, the correlation coefficient between the three dimensions was 0.462–0.664, and the Cronbach's α coefficient of each dimension was above 0.8, indicating good reliability and validity.

### Survey methods

This research represents a cross-sectional descriptive study using a questionnaire survey. After the informed consent forms of the research participants had been received, two professionally trained researchers distributed questionnaires to the patients and explained its content, the purpose of the study, and how to complete the questionnaire. If any issues regarding the method in which the questionnaire had been completed or concerning its content arose, the researcher would immediately return it to the participant after the latter had completed it for correction.

In this study, the sample size was estimated according to the influencing factors concerning fear of relapse in elderly patients with CHD. According to the empirical formula for multivariate analysis sample-size estimation, sample size (N) = number of study factors (n) × (10 ~ 15); 12 study factors were included in the multivariate analysis, considering a 20% sample attrition rate. Based on the results, the sample size required 144 to 216 cases. A total of 216 questionnaires were subsequently distributed and 200 valid questionnaires were recovered (effective recovery rate, 92.59%).

### Statistical methods

The SPSS Statistics 24.0 software program was used for data entry and analysis. The count data were described by frequency and percentage, and the measurement data conforming to the normal distribution were described by s. The effect of general data on the fear of recurrence was analysed by t-test and variance analysis, and the correlation between coping style, social support and fear of recurrence was analysed using Pearson’s correlation. Taking the total score of fear of recurrence as the dependent variable, the variables with statistical significance in the univariate and correlation analyses were included as independent variables in the multiple linear regression equation; then, the multiple linear regression analysis was carried out, and *P* < 0.05 was considered to be statistically significant.

## Results

### General information on elderly patients with coronary heart disease

The ages of the 200 patients ranged from 60.04 to 84.32 years; 64.00% (n = 128) were male, and 36.00% (n = 72) were female, and 156 cases (78%) were married. A total of 133 patients (66.50%) had a primary school education or lower, 52 (26.00%) had a secondary school education, and 15 (7.50%) had a college degree or higher. Among the patients, 41.8% (n = 53) lived in a village and worked in agriculture (40.2%, n = 51). The majority (87.2%, n = 111) of the patients were retired or on sick leave. Approximately 40.5% (n = 81) of the patients had a monthly household income per person below ¥1,000.

### The fear of progression questionnaire–short form, coping style and social support scores

The total FoP-Q-SF score of middle-aged and elderly CHD patients in this study was (38.46 ± 8.13) points; 119 cases (59.5%) scored higher than 34 points. The score of the physical health dimension was (19.89 ± 3.95), and the score for the social and family dimension was (18.57 ± 4.18) points. The positive coping style score was (21.60 ± 7.56) points, and the negative coping style score was (16.19 ± 6.78) points. The total SSRS score was (34.89 ± 9.83), including objective support (8.16 ± 2.99), subjective support (19.83 ± 4.67), and support utilisation (6.90 ± 2.47).

### Univariate analysis of fear of disease recurrence in elderly coronary heart disease patients

The descriptive statistics and correlations between demographic data, clinical characteristics and the fear of recurrence analysis are presented in Table 1. Marital status, educational level, monthly income of the participant, disease duration, recurrence rate, and knowledge of CHD were statistically significantly associated with participants’ fear levels (*P* < 0.05). Age, gender, the previous occupation of patients, and insurance status indicated no correlation with fear scores.

**Table 1:** Univariate analysis of FoP-Q-SF scores in elderly CHD patients (n = 200)

Items	n(%)	FoP-Q-SFscores ($\overline{x} \pm s$)	*t/F*	*P*
Age(years)			2.632^a^	0.064
60 ~ 70	118 (59.00)	38.94 ± 8.40		
> 70	82 (41.00)	37.98 ± 7.86		
Gender			− 1.821^a^	0.125
Male	128 (64.00)	38.90 ± 8.45		
Female	72 (36.00)	38.02 ± 7.81		
Marital status			5.178^a^	0.003
Married	156 (78.00)	39.90 ± 8.78		
No spouse	44 (32.00)	37.02 ± 7.48		
Educational level			3.397^b^	0.012
Elementary school and below	133 (66.50)	37.25 ± 7.45		
Junior high school and high school (or secondary school)	52 (26.00)	38.23 ± 7.98		
University and above	15 (7.50)	40.17 ± 8.89		
Personal monthly income (yuan)				
< 1000	81 (40.50)	36.41 ± 6.78	3.210^b^	0.015
1000 ~ 3000	91 (45.50)	38.56 ± 7.98		
> 3000	28 (14.00)	40.48 ± 8.90		
Previous occupation			− 1.298^a^	0.491
Mental work	27 (13.50)	38.95 ± 8.44		
Manual labor	81 (40.50)	37.96 ± 7.98		
Half-brain, half-manual work	92 (46.00)	38.47 ± 8.15		
Disease duration (years)			15.121^b^	0.001
< 1	6 (3.00)	41.23 ± 8.89		
1 ~	48 (24.00)	38.87 ± 8.59		
5 ~	90 (45.00)	36.01 ± 7.58		
> 10	56 (28.00)	35.44 ± 7.00		
Number of disease recurrences (times)			21.769^b^	0.000
0	40 (20.00)	36.01 ± 6.78		
1	73 (36.50)	37.22 ± 7.45		
2	62 (31.50)	40.16 ± 8.98		
≥ 3	25 (12.50)	43.09 ± 9.36		
Medical insurance or new rural cooperative medical system			− 1.898^a^	0.091
Yes	177 (88.50)	37.95 ± 7.89		
No	23 (11.50)	38.97 ± 8.37		
Do you know the disease			6.352^a^	0.002
Yes	154 (77.00)	37.64 ± 7.53		
No	46 (23.00)	39.28 ± 8.73		

^a^*t* value

^b^*F* value

### Correlation analysis between fear of disease recurrence, coping style and social support in elderly patients with coronary heart disease

The results of the Pearson’s correlation analysis showed that the total FoP-Q-SF score of elderly patients with CHD negatively correlated with positive coping style and social support (r = –0.621, –0.413, both *P* < 0.001), and positively correlated with negative coping style (r = –0.621, –0.413, both *P* < 0.001). = 0.232, *P* < 0.001) (see Table 2).

**Table 2:** Correlation between fear of disease recurrence and coping style and social support in elderly CHD patients

Variables	Fear of disease recurrence	
	*r*	*P*
Positive Coping	− 0.621	< 0.001
Negative Coping	0.232	< 0.001
Social support	− 0.413	< 0.001

### Multiple stepwise linear regression analysis of the factors influencing the fear of disease recurrence in elderly patients with coronary heart disease

The variables with statistical significance in the univariate and Pearson correlation analyses were subjected to multiple stepwise regression analyses (α in = 0.05, α out = 0.10), and the assignments are shown in Table 3. The results showed that the course of disease, the number of disease recurrences, active coping and social support entered the regression equation (see Table 4).

**Table 3:** Assignment methods of independent variables

Independent variable	Assignment method
Disease duration (years)	< 1 = 1; 1 ~ 4 = 2; 5 ~ 9 = 3; > 10 = 4
Number of disease recurrences (times)	0 = 1; 1 = 2; 2 = 3; ≥ 3 = 4
Positive Coping(scores)	Substitute the original value
Social support(scores)	Substitute the original value

**Table 4:** Multivariate analysis of factors influencing the fear of disease recurrence in elderly CHD patients (n = 200)

Variables	*B*	*SE*	*β*	*t*	*P*
Constant	16.911	2.044	–	9.121	0.000
Disease duration	− 1.388	0.389	− 0.145	− 3.344	0.001
Number of disease recurrences	0.555	0.116	0.167	3.549	0.000
Social support	− 0.430	0.026	− 0.283	− 4.544	0.000
Positive Coping	− 0.291	0.044	− 0.346	− 5.494	0.000

Note: Adjust R2 = 0.589, F = 32.397, *P* < 0.001

## Discussion

### The current situation of fear of recurrence in elderly coronary heart disease patients

The results showed that the FoP-Q-SF scores of elderly patients with CHD were (38.46 ± 8.13) points, and more than half of them had recurrence fear beyond the normal level (≥ 34 points). The China Cardiovascular Disease Report (2017) stated the number of cardiovascular patients in China as being 290 million, 11 million of which are CHD, with an age distribution that ranged from 55 to 79 years old.

As a common underlying disease in the elderly population, CHD has the characteristics of high recurrence. Therefore, elderly CHD patients also have a higher level of fear of disease recurrence. According to a survey, the recurrence rate of CHD patients in the community is as high as 40% [21]. Disease recurrence not only causes significant physical and mental pain for patients, but also leads to an increased burden on them, a waste of social health resources, and ‘difficulty seeing a doctor’. Medical staff should assess the fear of disease recurrence among elderly CHD patients promptly, identify high-risk patients and implement corresponding intervention measures to reduce the level of fear of disease recurrence.

### Analysis of the influencing factors of fear of recurrence in elderly coronary heart disease patients

#### Course of the disease

The results of this study showed that the shorter the duration of the disease, the higher the level of fear of disease recurrence (*P* < 0.05). In this study, the FoP-Q-SF score of patients with a course of disease < 1 year was much higher than that of patients with a course of disease ≥ 1 year. The reason for this may have been related to the strong emotional impact on patients who were newly diagnosed, difficulty accepting the facts of the disease, and often being immersed in negative emotions. Patients with a longer course of CHD are more likely to gradually experience a reduced fear of disease recurrence as they come to better understand their illness; accordingly, their FoP-Q-SF score will be lower than those of more recently diagnosed patients. The conclusions of some foreign studies [22, 23] contradict this, however. Therefore, the relationship between newly diagnosed patients, long-term survivors and the severity of fear of disease recurrence requires additional research.

#### The number of disease recurrences

The more recurrences the patient experienced, the higher their level of fear of recurrence. was The psychological and physical burdens, as well as the prognosis and rehabilitation problems brought on by recurrence of the disease increase the painful experience of the patient, which, in turn, creates fear of recurrence. Concurrently, after obtaining more knowledge about the disease, the patient learns of the serious consequences of repeated CHD recurrence, which will also cause them to be fearful of this happening. It is suggested that medical staff guide patients to correctly perceive the recurrence of the disease, help them to adjust unhealthy lifestyles, control the risk factors of CHD recurrence, monitor blood lipid levels, create a multidisciplinary comprehensive management mechanism, and improve patients’ self-management capabilities. The hospital–community–family follow-up method was adopted, and the patients were prescribed a healthy diet, exercise, medicine, psychological assistance, and rehabilitation guidance. This disease management and control approach was implemented to help prevent the recurrence of CHD.

#### Active response

The results of this study showed that for coping style, negative coping was positively correlated with recurrent fear (*P* < 0.05), and positive coping was negatively correlated with recurrent fear (*P* < 0.05), suggesting that the more positive the patient, the less likely they were to fear disease recurrence. ‘Coping style’ refers to the conscious, purposeful behaviour and cognitive style that people adopt when their environment changes. The positive coping style is mainly manifested through patients diverting their attention through positive activities, or by choosing to talk to others, or finding different ways in which to solve problems. Positive coping can help patients to improve their mood, face difficulties, and solve problems, thereby helping to improve negative emotions. Studies have shown that patients who adopted negative coping styles, such as smoking, drinking, and avoidance behaviour, experienced significantly higher levels of fear of recurrence than those who adopted active coping styles [24]. Medical staff should remain informed about the patient's current psychological status, and guide them to adopt positive coping methods to relieve psychological pressure and reduce the level of fear of disease recurrence.

#### Social support

The results of this study showed that the SSRS scores of elderly CHD patients negatively correlated with the fear of disease recurrence (r = –0.413, *P* < 0.05), indicating that the higher the SSRS score for elderly patients with CHD, the lower the level of fear of disease recurrence. ‘Social support’ refers to the spiritual or material support that an individual receives from the external environment, e.g. family, colleagues, friends, and their community when faced with a stressful event. Good social support can help patients effectively deal with economic, psychological and other factors. As an important external resource for elderly CHD patients, social support is particularly important for improving psychological conditions. In clinical nursing practice, medical staff should know more about the inner needs of patients, provide targeted information and psychological support, and encourage patients to relieve anxiety, fear and other negative emotions by communicating with family members and/or friends.

As a buffer factor for those who experience stressful events, social support can protect patients under stress and help them to maintain a positive emotional state of mind. Good social support is conducive to the recovery of the patient's physical and mental health and to overcome negative emotions.

## Conclusion

This study describes the current situation and factors of fear regarding CHD recurrence in a Chinese population cohort. This is a common issue in China and internationally. The severity of this type of fear among Chinese elderly patients may vary, as family factors play an important role in Chinese culture and often include wider family members where an extensive network of relationships may enhance family resilience and social support. [25] To clarify these factors, the study employed questionnaires that included items from the Disease Progression Simplified Scale, the SCSQ, and the SSRS to score and analyse the fear of recurrence.

The results of this study showed that elderly patients with CHD had a higher level of fear of CHD recurrence, which was related to the course of the disease, the number of previous disease recurrences, coping styles and social support levels. Medical staff should provide psychological support and health education according to individual characteristics to help patients correctly understand. To reduce the fear of recurrence, the patient's social support system should also be reviewed and their family should be encouraged to provide patients with additional care.

## Limitations

Despite the supportive findings of this study, several limitations must be acknowledged, such as its small sample size and a lack of multi-centre samples from different hospitals. Due to limitations on the study conditions, the scoring criteria for complications among elderly patients, such as diabetes, obesity and hypertension, were not included. It is recommended to carry out large-scale, multi-centre collaborative research in the future to make the research results more representative. Meanwhile, attempts were made to further quantify the degree of fear of disease recurrence in elderly patients to provide a basis for the subsequent formulation of effective intervention plans.

## Data Availability

The datesets used or analyzed during the current study are available from the corresponding author on reasonable request.
