# Correction to: Fear of recurrence in elderly patients with coronary heart disease: the current situation and influencing factors according to a questionnaire analysis

**DOI:** 10.1186/s12872-023-03297-6

**Published:** 2023-05-19

**Authors:** Jing Zhen, Jing Wang, Yi‑Lin Wang, Jin Jiao, Jing Li, Xiao‑Jing Du, Yan‑Ling Li

**Affiliations:** 1 Office of Infection and Control, Affiliated Hospital of Hebei University, Baoding, China; 2 Inspection department, Affiliated Hospital of Hebei University, Baoding, 071000 Hebei China; 3 School of Basic Medicine of Shandong University, Jinan, 250012 Shandong China; 4 Medical Oncology Department, Affiliated Hospital of Hebei University, Baoding, China; 5 Cardiovascular Department, Affiliated Hospital of Hebei University, Baoding, China; 6 School of Nursing, Hebei University, Baoding, China; 7 Department of Tuberculosis, Affiliated Hospitalof Hebei University, Baoding, China

**Correction to: BMC Cardiovascular Disorders**

10.1186/s12872-022-02853-w

Following publication of the original article [1], the affiliations of the authors Jing Zhen, Jin Jiao and Jing Li has been updated has shown below:

^1^ Office of Infection and Control,Affiliated Hospital of Hebei University, Baoding, China.

^2^ Inspection department, Affiliated Hospital of Hebei University, Baoding 071000, Hebei, China.

^3^ School of Basic Medicine of Shandong University,Jinan 250012, Shandong, China.

^4^ Medical Oncology Department, Affiliated Hospital of Hebei University, Baoding, China.

^5^ Cardiovascular Department,Affiliated Hospital of Hebei University, Baoding, China. ^6^ School of Nursing, Hebei University, Baoding, China.

^7^ Department of Tuberculosis, Affiliated Hospitalof Hebei University, Baoding, China.

The original article has been corrected.

